# Advisory Committee on Immunization Practices Recommended Immunization Schedule for Adults Aged 19 Years or Older — United States, 2018

**DOI:** 10.15585/mmwr.mm6705e3

**Published:** 2018-02-09

**Authors:** David K. Kim, Laura E. Riley, Paul Hunter

**Affiliations:** ^1^Immunization Services Division, National Center for Immunization and Respiratory Diseases, CDC; ^2^Harvard University, Cambridge, Massachusetts; ^3^University of Wisconsin, Madison, Wisconsin.

In October 2017, the Advisory Committee on Immunization Practices (ACIP) voted to approve the Recommended Immunization Schedule for Adults Aged 19 Years or Older, United States, 2018. The 2018 adult immunization schedule summarizes ACIP recommendations in two figures and a table of contraindications and precautions for vaccines recommended for adults, and is intended is to assist health care providers in implementing the current ACIP recommendations for vaccinating adults. The schedule can be found at https://www.cdc.gov/vaccines/schedules.* The full ACIP recommendations for each vaccine are available at https://www.cdc.gov/vaccines/hcp/acip-recs/index.html. The 2018 adult immunization schedule has also been approved by the American College of Physicians (https://www.acponline.org), the American Academy of Family Physicians (https://www.aafp.org), the American College of Obstetricians and Gynecologists (https://www.acog.org), and the American College of Nurse-Midwives (http://www.midwife.org). The ACIP-recommended use of each vaccine is developed after an in-depth review of vaccine-related data, including data on disease epidemiology, vaccine efficacy and effectiveness, vaccine safety, feasibility of program implementation, and economic aspects of immunization policy (*1*).

The adult immunization schedule also contains information on general principles of immunization for adults; considerations for special populations, such as pregnant women; reference resources pertinent to adult immunization; instructions for reporting adverse events associated with vaccinations and suspected cases of reportable vaccine-preventable diseases; and an ACIP-approved list of standardized abbreviations for vaccines recommended for adults. The two figures in the adult immunization schedule are accompanied by footnotes that provide important details on vaccination recommendations, such as the number of doses in a vaccination series and dosing intervals. Health care providers are advised to use the figures and the footnotes together. Changes in the 2018 adult immunization schedule from the previous year’s schedule include new ACIP recommendations for the use of recombinant zoster vaccine (RZV) for adults aged 50 years or older and the use of an additional dose of measles, mumps, and rubella vaccine (MMR) in a mumps outbreak setting.

## Changes in the 2018 Adult Immunization Schedule

**Zoster Vaccination** (*2*). On October 20, 2017, the Food and Drug Administration approved the use of RZV (SHINGRIX, GlaxoSmithKline [GSK]) for adults aged 50 years or older for the prevention of herpes zoster (shingles) and its complications. On October 25, ACIP recommended the use of 1) RZV among immunocompetent adults aged 50 years or older for the prevention of herpes zoster and related complications, 2) RZV among adults aged 50 years or older who previously received the zoster vaccine live (ZVL) (ZOSTAVAX, Merck and Co.), and 3) either RZV or ZVL for adults aged 60 years or older (RZV is preferred). On October 26, 2017, ACIP recommended the following in the 2018 adult immunization schedule:

- Administer 2 doses of RZV 2–6 months apart to adults aged 50 years or older regardless of past episode of herpes zoster or receipt of ZVL.
- Administer 2 doses of RZV 2–6 months apart to adults who previously received ZVL at least 2 months after ZVL.
- For adults aged 60 years or older, administer either RZV or ZVL (RZV is preferred).

The clinical trials for RZV excluded pregnancy and confirmed or suspected immunocompromising conditions that can result from disease (e.g., malignancy, HIV infection) or therapy (e.g., cancer chemotherapy, treatment for autoimmune disorders) (*3*–*6*). Therefore, no ACIP recommendation currently exists for use of RZV among pregnant women (health care providers should consider delaying administration of RZV for pregnant women) or adults with immunocompromising conditions, including HIV infection (additional discussions and recommendations by ACIP on the use of RZV in adults with immunocompromising conditions are pending).

Consistent with the existing recommended use of ZVL, ACIP recommended RZV for adults who are receiving low-dose (<20 mg/day of prednisone or equivalent) or short-term (<14 days of corticosteroids) immunosuppressive therapy, are anticipating immunosuppression, or have recovered from an immunocompromising illness (*7*). The clinical trials for RZV did not exclude adults with non-immunocompromising chronic health conditions (*3*–*6*). Therefore, given the safety and effectiveness profiles of other conjugate vaccines recommended for adults (e.g., hepatitis B and pneumococcal vaccines), ACIP recommended that RZV should routinely be used for age-eligible adults with diabetes mellitus; chronic heart, lung, liver, or kidney disease; functional or anatomical asplenia; or complement deficiencies.

**MMR Vaccination** (*8*). On 25 October, ACIP updated MMR vaccination recommendations to include the use of a third dose of a mumps virus–containing vaccine for persons previously vaccinated with 2 doses of a mumps virus–containing vaccine who are identified by public health authorities as being a part of a group or population at risk for acquiring mumps because of an outbreak. During a mumps outbreak, persons identified as being at increased risk and who have received ≤2 doses of mumps virus–containing vaccine or whose mumps vaccination status is unknown should receive 1 dose of MMR. This change is described in the 2018 adult immunization schedule as follows:

- Administer 1 dose of MMR to adults who previously received ≤2 doses of mumps virus–containing vaccine and are identified by a public health authority to be at increased risk during a mumps outbreak.

Adults without evidence of immunity to mumps (defined as birth before 1957, documentation of receipt of MMR, or laboratory evidence of immunity or disease) are routinely recommended to receive 1 dose of MMR for mumps prevention. However, students in postsecondary educational institutions, international travelers, or household contacts of immunocompromised persons should receive 2 doses of MMR at least 28 days apart. In a mumps outbreak setting, those adults identified by a public health authority to be at risk should receive 1 dose of MMR regardless of whether they previously received 0, 1, or 2 doses of a mumps virus–containing vaccine.

**Notable Changes to Figures 1 and 2.** The footnotes in the 2018 adult immunization schedule should be used in conjunction with “Figure 1. Recommended immunization schedule for adults aged 19 years or older, by age group” and “Figure 2. Recommended immunization schedule for adults aged 19 years or older by medical condition and other indications.” The footnotes contain additional general information (e.g., dosing intervals for vaccination series) and considerations for special populations (e.g., pregnant women, adults with HIV infection). The footnotes in the adult immunization schedule and the child and adolescent immunization schedule have been harmonized to be more consistent with one another (*9*). Notable changes in Figures 1 and 2 include the following:

- In Figures 1 and 2, “ZVL” replaced the term “HZV” (herpes zoster vaccine) that was used in past adult immunization schedules to refer to the live zoster vaccine. A row for RZV was added above the row for ZVL, and a dashed line was used to separate RZV and ZVL rows to denote that the two zoster vaccines are recommended for the same purpose. In the indication bars for RZV, the text stating that RZV is preferred over ZVL has been added when either RZV or ZVL can be used for adults aged 60 years or older.
- In Figures 1 and 2, “Td/Tdap” (tetanus and reduced diphtheria toxoids/tetanus and reduced diphtheria toxoids and acellular pertussis vaccine) has been replaced by “Tdap or Td,” and the text in the indication bar has been revised to “1 dose Tdap, then Td booster every 10 years.” 1 dose of Tdap is recommended for adults who have not previously received Tdap as an adult or child (1 dose of Tdap is routinely recommended at age 11–12 years), except for pregnant women, for whom 1 dose of Tdap is recommended in each pregnancy during the early part of gestational weeks 27–36.
- In Figures 1 and 2, the text in the indication bar for MenACWY (serogroups A, C, W, and Y meningococcal vaccine) has been revised to “1 or 2 doses depending on indication, then a booster dose every 5 years if risk remains.” Adults with functional or anatomical asplenia, persistent complement component deficiencies, or HIV infection should receive 2 doses of MenACWY and be revaccinated every 5 years. One dose of MenACWY is recommended for microbiologists who work with isolates of *Neisseria meningitidis* and travelers in countries with endemic or epidemic meningococcal disease, and a booster dose of MenACWY is indicated every 5 years if the risk remains. One dose of MenACWY is recommended for first-year college students living in residence halls and military recruits. MPSV4 (4-valent meningococcal polysaccharide vaccine) is no longer available and has been removed from the adult immunization schedule.
- In Figure 1, the text in the indication bar for MMR has been changed to “1 or 2 doses depending on indication (if born in 1957 or later).” One dose of MMR is routinely recommended for adults born in 1957 or later who do not have evidence of immunity to measles, mumps, or rubella. However, for students in postsecondary educational institutions, international travelers, and household contacts of immunocompromised persons, 2 doses of MMR administered at least 28 days apart are routinely recommended.
- In Figure 1, the text in the indication bars for human papillomavirus (HPV) vaccine for females and males (*10*) has been revised to “2 or 3 doses depending on age at series initiation.”

## More Information

Details on these updates and information on other vaccines recommended for adults are available online under Adult Immunization Schedule, United States, 2018, at https://www.cdc.gov/vaccines/schedules/hcp/adult.html and in the Annals of Internal Medicine (*11*). The full ACIP recommendations for each vaccine are also available online at https://www.cdc.gov/vaccines/hcp/acip-recs/index.html.

## Footnotes

* CDC encourages organizations that previously have relied on copying the adult immunization schedule on their websites to use syndication instead, as a more reliable method for displaying the most current and accurate adult immunization schedule. Use of content syndication requires a one-time step that ensures an organization's website displays the adult immunization schedule as soon as it is published or revised. The syndication code for the adult immunization schedule and instructions for its use can be found at https://www.cdc.gov/vaccines/schedules/syndicate.html. Requests for technical assistance for adult immunization schedule syndication can be sent to ncirdwebteam@cdc.gov.
